# The pan-cancer landscape of crosstalk between epithelial-mesenchymal transition and immune evasion relevant to prognosis and immunotherapy response

**DOI:** 10.1038/s41698-021-00200-4

**Published:** 2021-06-22

**Authors:** Guangyu Wang, Dandan Xu, Zicheng Zhang, Xinhui Li, Jiaqi Shi, Jie Sun, Huan-Zhong Liu, Xiaobo Li, Meng Zhou, Tongsen Zheng

**Affiliations:** 1 Department of Gastrointestinal Medical oncology, Harbin Medical University Cancer Hospital, Harbin, PR China; 2 School of Biomedical Engineering, School of Ophthalmology & Optometry and Eye Hospital, Wenzhou Medical University, Wenzhou, PR China; 3 Centre for Addiction and Mental Health, Toronto, ON Canada; 4 Department of Psychiatry, University of Toronto, Toronto, ON Canada; 5 College of Bioinformatics Science and Technology, Harbin Medical University, Harbin, PR China; 6 Chaohu Hospital of Anhui Medical University, Hefei, Anhui PR China; 7 Department of Pathology, Harbin Medical University, Harbin, PR China; 8 Department of Phase 1 Trials Center, Harbin Medical University Cancer Hospital, Harbin, PR China

**Keywords:** Cancer microenvironment, Tumour biomarkers

## Abstract

An emerging body of evidence has recently recognized the coexistence of epithelial-mesenchymal transition (EMT) and immune response. However, a systems-level view and survey of the interplay between EMT and immune escape program, and their impact on tumor behavior and clinical outcome across various types of cancer is lacking. Here, we performed comprehensive multi-omics analyses to characterize the landscape of crosstalk between EMT and immune evasion and their clinical relevance across 17 types of solid cancer. Our study showed the presence of complex and dynamic immunomodulatory crosstalk between EMT and immune evasion shared by pan-cancer, and the crosstalk was significantly associated with cancer prognosis and immunotherapy response. Integrative quantitative analyses of genomics and immunogenomics revealed that cellular composition of immune infiltrates, non-synonymous mutation burden, chromosomal instability and oncogenic gene alterations are associated with the balance between EMT and immune evasion. Finally, we proposed a scoring model termed EMT-CYT Index (ECI) to quantify the EMT-immunity axis, which was a superior predictor of prognosis and immunotherapy response across different malignancies. By providing a systematic overview of crosstalk between EMT and immune evasion, our study highlights the potential of pan-cancer EMT-immunity crosstalk as a paradigm for dissecting molecular mechanisms underlying cancer progression and guiding more effective and generalized immunotherapy strategies.

## Introduction

Epithelial-mesenchymal transition (EMT) is a transformative process of cells in which they transition from an epithelial form to a mesenchymal form, and this serves a critical role in embryogenesis and tissue repair.^1^ EMT is also involved in human malignancies and drives cancer progression.^2^ The most aggressive subtypes of malignant epithelial tumors, such as basal-like breast cancer with higher rates of metastasis, usually show a higher level of EMT.^3^ Moreover, during EMT, apoptosis is suppressed in cancer cells,^4^ and this facilitates the induction of cancer stem cells,^1,5,6^ stimulates angiogenesis^7^ and enhances immunosuppression^8^ in the tumor microenvironment. Additionally, successful EMT is a common characteristic observed in the disseminated tumor cells and circulating tumor cells, which contributes to distant metastasis of cancer.^9–11^ Thus, the degree of EMT is positively correlated with a less favorable outcome in patients with cancer.^1,2^

The preclinical and clinical data suggest that EMT is a common phenomenon during or after therapy, and therapy-induced EMT implies therapy resistance and cancer progression.^12,13^ Radiotherapy,^14,15^ chemotherapy based on platinum,^16^ anti-metabolites^17^ or alkylating agents^4^ have been shown to induce EMT, and emerging evidence has shown that EMT in the post (neoadjuvant) chemotherapy or radiotherapy specimen is correlated with relapse or shorter survival times.^14,15,18^ Recently, targeted therapy has also been shown to induce EMT. For example, anti-human epidermal growth factor receptor therapy induces EMT in breast cancer and gastric cancer,^19,20^ whereas anti-anaplastic lymphoma kinase and anti-epidermal growth factor therapy induce EMT in lung carcinoma.^21,22^ Moreover, anti-angiogenesis therapy is also reported to induce EMT.^23^ These studies suggest that conventional therapies induce EMT across a broad spectrum of cancer types. However, research into the association between EMT and immunotherapy, a promising therapeutic strategy for cancer that aims to increase the cytolytic activity of infiltrated immune cytolytic cells, is limited.

Typically, it is widely suggested that the infiltration of cytolytic immune cells, such as CD8+ T cells, predicts a beneficial prognosis in multiple types of cancer by directly killing cancer cells.^24^ However, it has been shown that CD8+ T cells are involved in cancer relapse by directly inducing EMT-mediated immunoediting in a mouse breast cancer model.^25^ Additionally, Alsuliman et al. demonstrated the association between EMT and PD-L1-mediated immune evasion in breast cancer cells.^26^ A recent study also showed that a high degree of immune infiltration is strongly associated with the induction of EMT in lung adenocarcinoma, which in turn is correlated with increased expression of suppressive immune checkpoints, including CTLA-4 and PD-L1.^8^ However, although the coexistence of EMT and immune evasion were observed in several types of cancer, a pan-cancer landscape-based analysis of crosstalk between EMT and immune evasion, and their clinical implications for immunostimulatory therapies has not been established, which remains a fundamental challenge.

In the present study, to improve our understanding of EMT and immune evasion interactions, we performed a pan-cancer multi-omics analysis to characterize immunomodulatory crosstalk between EMT and immune evasion by integrating multi-omics data of 17 types of solid cancer. Additionally, we systematically analyzed genomic and genetic characteristics that may influence the immunomodulatory crosstalk between EMT and immune evasion. Finally, we investigated the clinical impact of EMT and immune evasion crosstalk on cancer prognosis and immunotherapy response and proposed a scoring model to predict prognosis and response to checkpoint blockers for distinct types of cancer.

## Results

### Systematic analysis of immunomodulatory crosstalk between EMT and immune evasion

**Fig. 1:**
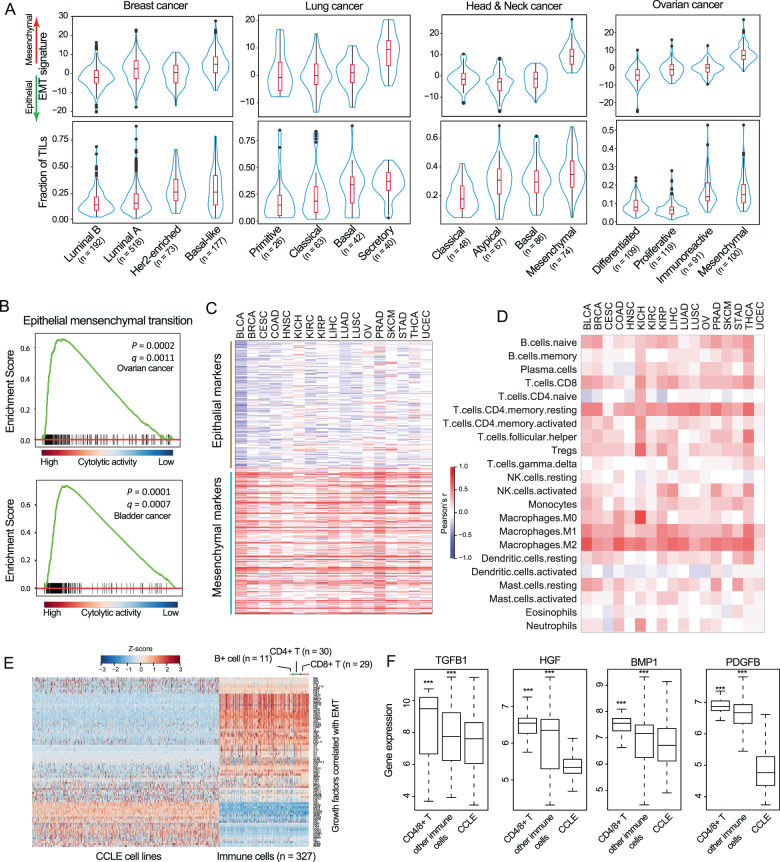
Pan-cancer landscapes of crosstalk between EMT and immune evasion across 17 types of solid cancer. **A** Violin plots of the infiltration levels of TILs and EMT levels for different subtypes of four types of cancer. **B** Gene set enrichment analysis of CYT_high_ and CYT_low_ tumors in ovarian cancer and bladder cancer. **C** Correlation between expression of mesenchymal and epithelial marker genes and CYT across the different types of cancer. **D** Correlation between the infiltration levels of the 22 immune cell subpopulations and EMT scores across different types of cancer. **E** Expression heatmaps of 83 EMT-associated growth factors in cancer cell lines and immune cell lines. **F** Boxplots of expression of *TGFB1*, *HGF*, *BMP1* and *PDGFB* in different cell groups. ***P < 0.001. CYT cytolytic activity, EMT epithelial-mesenchymal transition, TILs tumor-infiltrating leukocytes.

We first investigated the relationship between tumor-infiltrating lymphocytes (TILs) and EMT features in pan-cancer and found significant positive correlations across the 17 cancer types (Pearson correlation *r* = 0.372, *P* < 0.001; Supplementary Fig. 1A). We then tested whether this correlation confounded the identification of cancer subtypes, and found that cancer subtypes with a higher fraction of TILs exhibited a consistently higher EMT signature, even though there were notable discrepancies in the infiltration levels of TILs for different cancer subtypes (Fig. 1A). For example, the mesenchymal subtype of HNSC and OV showed the highest level of TILs, consistent with a previous study that showed that the low tumor purity found in tumors of mesenchymal subtypes confounded the subtype identification.^27^

It has previously been shown that various immune-suppressive cells are capable of inducing EMT through growth factors (such as TGFβ and TNFα).^28,29^ However, whether cytolytic immune cells are associated with EMT features has not been studied previously, to the best of our knowledge. We, therefore, investigated the potential biological processes associated with immune cytolytic activity (CYT) which was used to assessed immune evasion using the geometric mean of GZMA and PRF1 expression values.^30^ By dividing tumors into CYT_high_ and CYT_low_ groups for each cancer type, the results of GSEA revealed that immune-related pathways and EMT processes were significantly enriched in CYT_high_ tumors (Fig. 1B and Supplementary Fig. 1B). Additionally, CYT_high_ tumors tended to exhibit mesenchymal-like phenotypes with a higher EMT signature (Supplementary Fig. 1C). Furthermore, we observed that mesenchymal marker genes consistently showed a positive correlation with CYT across the 17 types of cancer, whereas epithelial marker genes typically exhibited a negative correlation with CYT (Fig. 1C).

Although CD8+ effector T cells have been shown to produce EMT-inducing growth factors, such as TGFβ,^31^ their relative importance compared with immune-suppressive cells has not been assessed. Therefore, we systematically characterized the associations of the cellular composition of the immune infiltrate with EMT features across the 17 different cancer types, and found that the infiltration of most immune cell subpopulations was positively correlated with EMT scores, including with the immune effector cells, such as B cells, CD8+ T cells and M1 macrophages (Fig. 1D). Then, we assembled 327 transcriptomes for 28 immune cell subpopulations and 83 EMT-associated growth factors and found that effector cell subpopulations expressed a similar set of growth factors as the immune suppressive cells when compared with cancer cell lines (Fig. 1E). Of the 83 EMT-associated growth factors, 55 growth factors were observed to not only reveal significantly higher expression in immune cells than cancer cell lines, but also showed a strong correlation with EMT, CYT and the fraction of TILs (Supplementary Fig. 1D). For example, four growth factors (TGFB1, HGF, BMP1 and PDGFB) that are well-known to induce EMT, exhibited higher expression levels in CD4/8+ T cells compared with other immune and tumor cells (Fig. 1F and Supplementary Fig. 1D), suggesting that anti-immune evasion promotes EMT as well by producing growth factors which induce EMT. Together, the above results demonstrate the existence of a pan-cancer correlation between immune evasion and EMT, and suggested that immune effector cells may be able to induce EMT to a similar degree as immune suppressive cells.

### Immunomodulatory crosstalk between EMT and immune evasion determines cancer prognosis

**Fig. 2:**
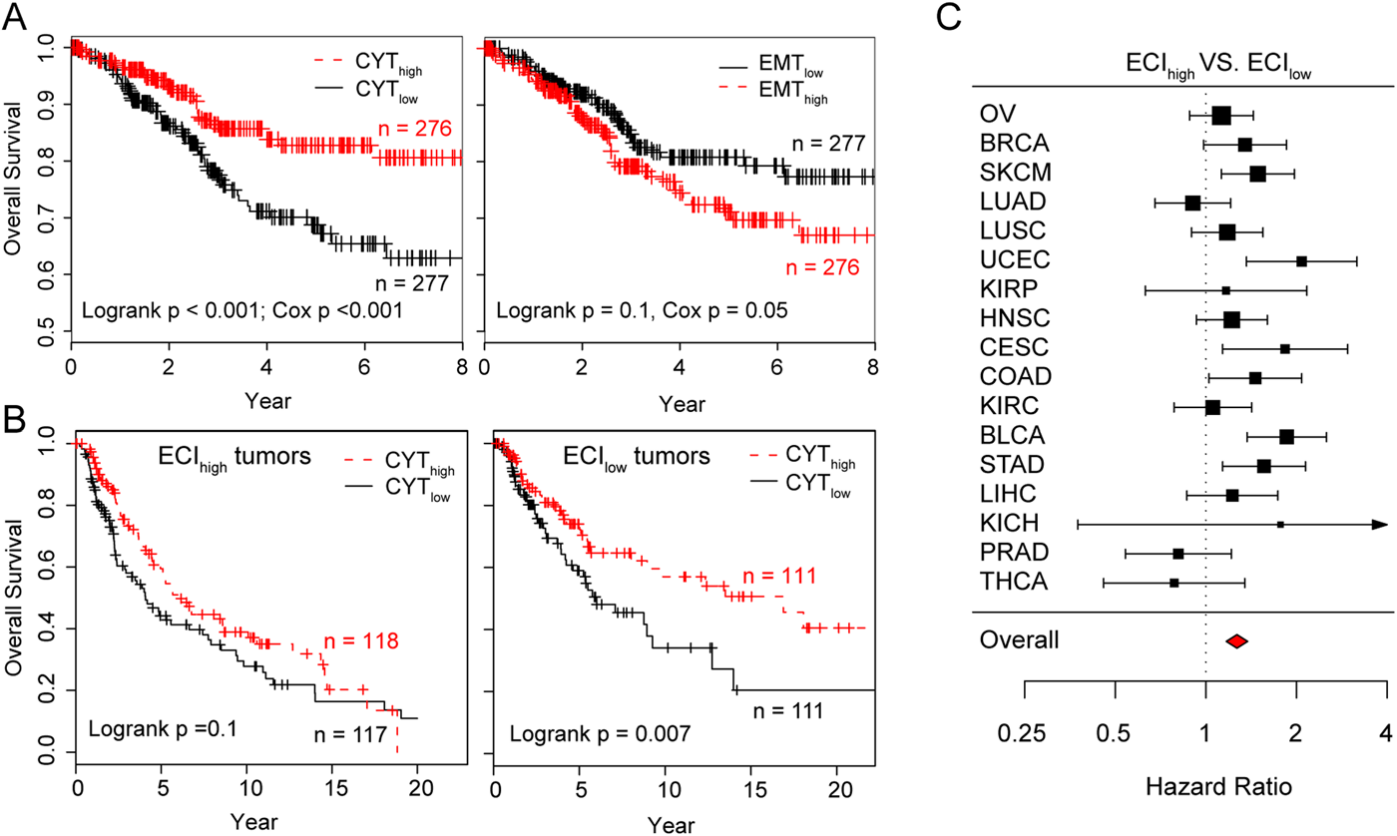
Prognostic association of EMT and immune evasion interactions. **A** Overall survival of UCEC patients according to CYT and EMT scores. Adjusted survival curves were generated using the multivariate Cox proportional hazards model. **B** Overall survival of ECI_high_ and ECI_low_ tumors patients according to CYT and EMT scores in melanoma. **C** Forest plot visualizing the hazard ratios (95% confidence intervals) of univariate Cox proportional regression analyses for ECI across 17 types of solid cancer. CYT cytolytic activity, EMT epithelial-mesenchymal transition, ECI EMT-CYT index, UCEC uterine corpus endometrial carcinoma.

Infiltrating immune effector cells are generally associated with improved survival and response to immunotherapy, whereas EMT is generally associated with poor outcomes and immune suppression. Using UCEC as an example, tumors with a higher CYT score were associated with more prolonged survival (Cox *P* < 0.001), whereas EMT was associated with poor survival (Cox *P* = 0.05) (Fig. 2A), although CYT and EMT were positively correlated with each other in UCEC. We also tested the above observation at a pan-cancer level using meta-analysis to leverage different cancer types and found that CYT scores were significantly associated with favorable outcomes [hazard ratio (HR) = 1.09, 95% confidence interval (CI): 1.01−1.19)], whereas EMT was significantly associated with worse survival (HR = 0.84, 95% CI: 0.78−0.91) (Supplementary Fig. 2), despite the positive correlation between these two factors.

The dilemma of observing two positively correlated factors inversely correlated with cancer outcome indicates that the crosstalk between EMT and immune evasion is likely more clinically relevant than either immune evasion or EMT alone. Therefore, we proposed an ECI signature to quantitatively model the crosstalk between immune evasion and EMT in the tumor microenvironment (TME), which estimates the extent of EMT deviation from the expected amount based on the corresponding CYT score in a tumor. Pan-cancer analysis using multivariate Cox proportional hazards model correcting for cancer types revealed a significant antagonistic interaction (Wald test, *P* = 0.002), indicating that a higher ECI will decrease the beneficial association between immune evasion and survival. An example of this is shown in Fig. 2B for melanoma, a higher CYT score was significantly associated with survival benefit only for ECI_low_ tumors (log-rank test, *P* < 0.01) (Fig. 2B). Additionally, ECI was a more accurate prognostic factor for predicting a worse survival outcome (HR = 1.27, 95% CI: 1.17–1.38) compared with either EMT or CYT alone in most of the cancer types assessed (Fig. 2C and Supplementary Fig. 2). These results suggest that a high infiltration of immune cells does not necessarily indicate an overall tumoricidal effect. For example, evidence from a previous clinical study demonstrated that although the mesenchymal subtype of OV exhibits a high CYT score, the mesenchymal subtype was associated with the worst clinical outcome.^32^

### Immunomodulatory crosstalk between EMT and immune evasion is associated with the response to immune checkpoint blockade

**Fig. 3:**
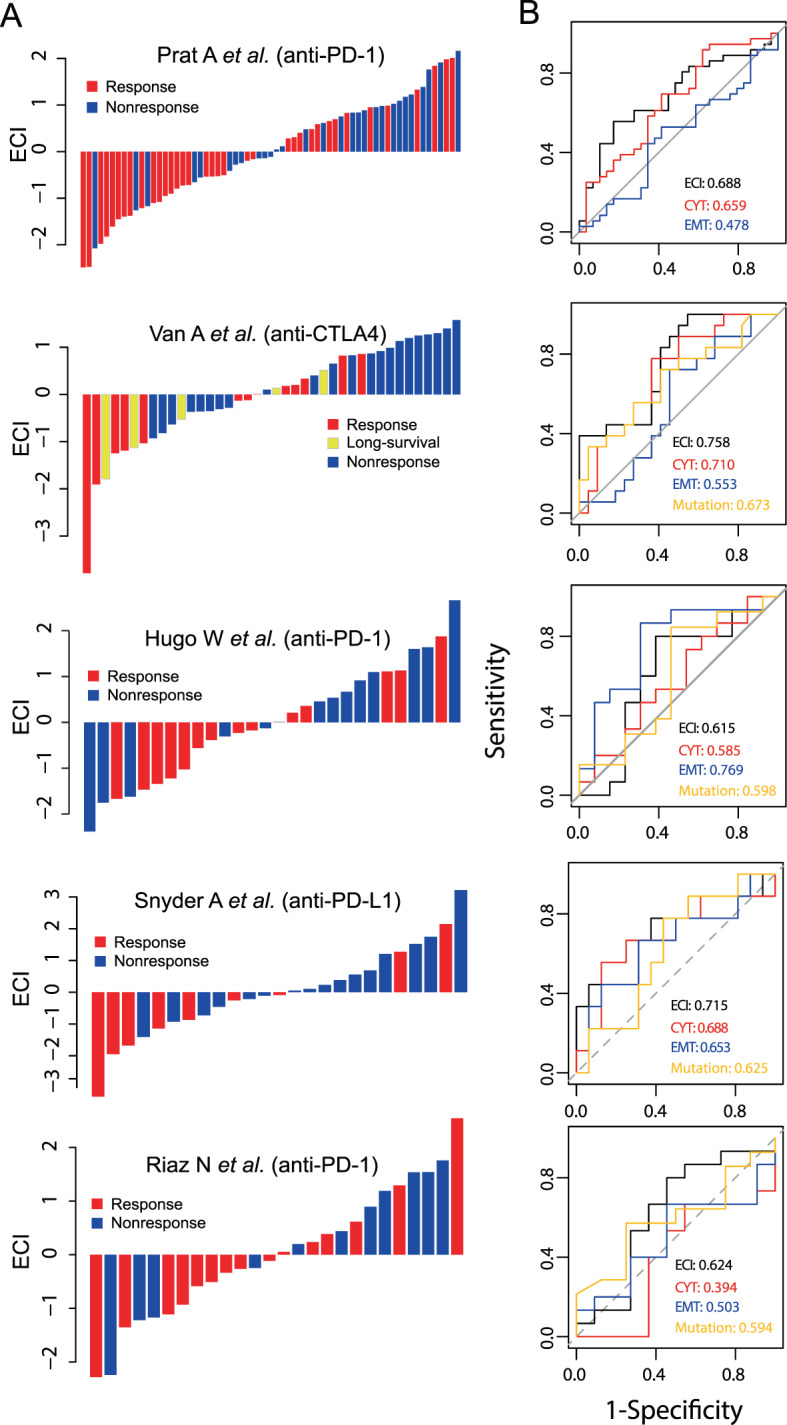
ECI predicts response to immune checkpoint blockade. **A** ECI and ICB response of patients in five independent immunotherapy datasets. Tumors were sorted according to their ECI. **B** Receiver operating characteristic curves for ICB response for the ECI, CYT, EMT and TMB of the five independent immunotherapy datasets. CYT cytolytic activity, EMT epithelial-mesenchymal transition, ECI EMT-CYT index, ICB immune checkpoint blockage, TMB tumor mutation burden.

Next, we investigated whether the EMT-immunity axis influenced the clinical immunotherapy response. As shown in Fig. 3A, tumors resistant to ICB were associated with an increase of ECI in five published immunotherapy datasets treated with ICB therapy (Fig. 3A). Overall, the response rate for ECI_low_ tumors was 60.3% (35/23) in all the immunotherapy datasets, whereas it was only 36.1% (22/39) for ECI_high_ tumors (Fisher’s exact test; *P* = 0.01). We further determined the performance of ECI in predicting ICB response. As shown in Fig. 3B, the ECI achieved consistently better predictive performance for ICB response with an area under the curve of 0.615–0.758 compared with the TMB (0.594–0.673), EMT signature (0.478–0.769), or CYT (0.394–0.710) across different immunotherapy datasets (Fig. 3B). TMB exhibited a modest predictive performance across the different datasets, whereas CYT was not predictive of ICB response in the Riaz et al. dataset, and EMT was not predictive in the Prat et al. dataset. A possible explanation for this is that ECI was used as a measure of crosstalk between EMT and CYT to evaluate the immune resistance, and thus has overall better performance. We further compared the distribution of EMT, CYT and ECI in cancers with high ICB response including CESC, BLCA, HNSC, LUSC and LUAD, with relatively low response including OV, PRAD and BRCA. As shown in Supplementary Fig. 3, For CESC, BLCA, HNSC, LUSC and LUAD, CYT is relatively higher than their EMT, whereas for OV, PRAD and BRCA, CYT is relatively lower than their EMT. Using ECI, we found that CESC, BLCA, HNSC, LUSC and LUAD have lower ECI values than OV, PRAD and BRCA. These results highlight the potential clinical significance of ECI for the identification of patients that will benefit from immunotherapy.

### Cellular composition of the immune infiltrates contributes to the crosstalk between EMT and immune evasion

**Fig. 4:**
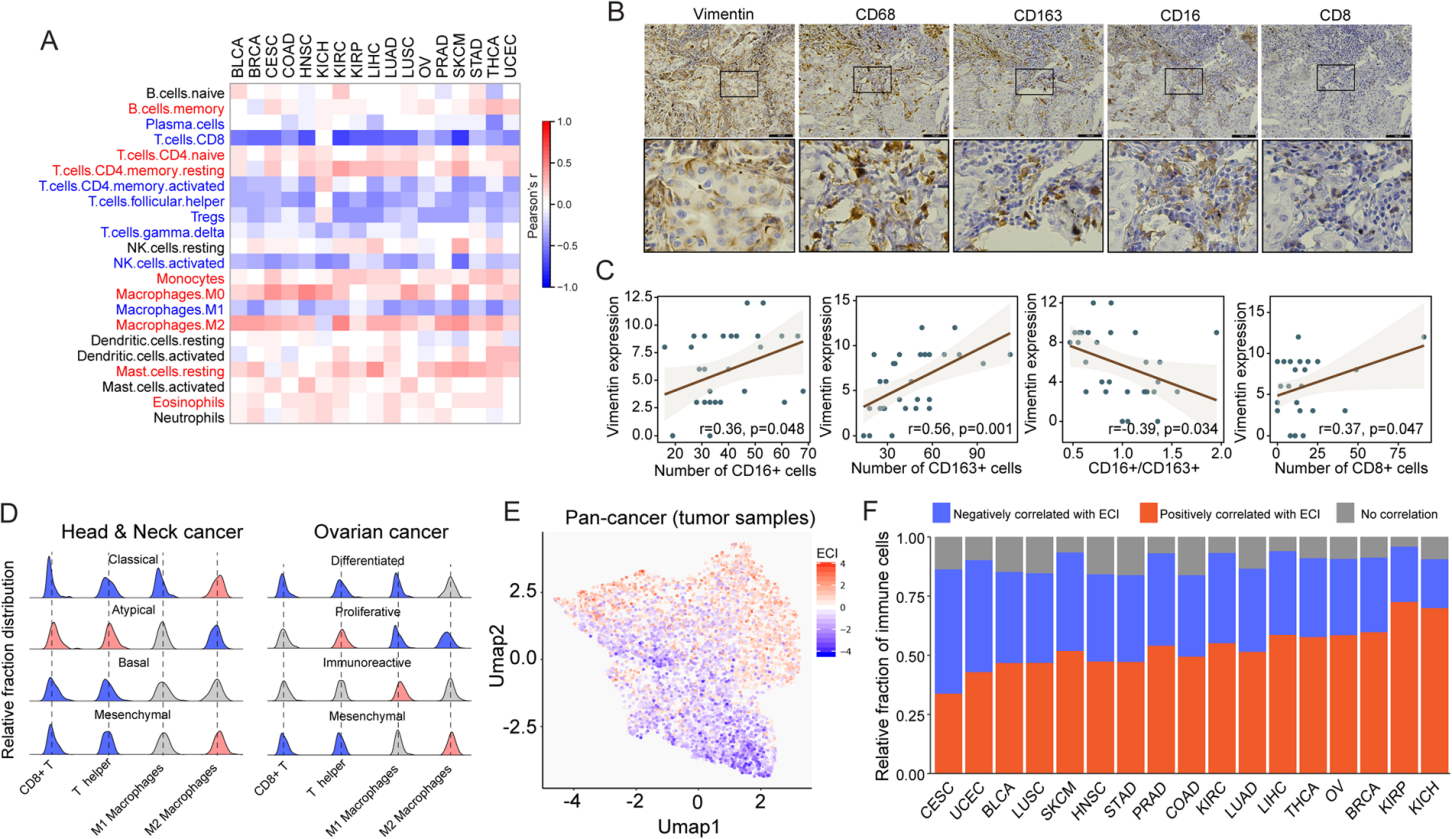
Association of the cellular composition of immune infiltrates with EMT and immune evasion interactions. **A** Correlation between ECI and the infiltration of 22 immune cell subpopulations across 17 types of solid cancer. **B** Immunohistochemistry assay was performed to mark macrophages or CTLs and detect the expression of Vimentin in lung cancer. **C** Pearson correlation analysis was used to assess the correlation between Vimentin expression and macrophages or CTLs infiltration. **D** Distribution of relative infiltration fraction of CD8+ T, T helper cells, M1 and M2 macrophages cells in different cancer subtypes of ovarian cancer and head and neck squamous cell carcinoma. **E** Unsupervised cluster of pan-cancer tumor samples using uniform manifold approximation and projection based on the relative infiltration fraction of the 22 immune cell subpopulations. **F** Relative fraction of infiltrating immune cells positively or negatively correlated with ECI across the 17 types of solid cancer. Tumors are ordered by the ratio of infiltrating immune cells negatively correlated with ECI (blue color) vs. infiltrating immune cells positively correlated with ECI (red color). ECI EMT-CYT index, EMT epithelial-mesenchymal transition.

Above, it was shown that almost all immune cells produce a similar set of growth factors and show a positive correlation with the EMT signature. However, immune evasion is determined by the relative composition of immune effector cells and suppressive cells. Therefore, we hypothesized that identifying the cellular composition of immune infiltrates is essential for understanding the crosstalk between immune evasion and EMT. To test this hypothesis, we investigate the association between ECI with a relative fraction of different immune cell types using the Pearson correlation analysis for each cancer type. We found that the infiltration of CD8+ T cells, activated memory CD4+ T Cells, regulatory T Cells (Tregs), plasma cells, T helper cells, ℽδ T cells, M1 macrophages and activated NK cells exhibited a significant negative association with ECI, whereas M0/M2 macrophages, monocytes, mast cells, eosinophils, memory B cells, naive CD4+ T cells and resting memory CD4+ T cells showed a significant positive association with ECI at the pan-cancer level (Fig. 4A). These results were consistent with the opposing effects of anti-immune evasion.^33^ For example, M1 macrophages was generally considered as pro-inflammatory and can promote anti-tumor immunity, whereas M2 macrophages generally promote cell proliferation and participate in EMT-associated processes, such as wound healing and tissue repair.^34^ As a case study, an immunohistochemistry assay was performed to mark macrophages or CD8+ T cells and detect the expression of vimentin in lung cancer. M1 expressed both CD68 and CD16, M2 expressed both CD68 and CD163. The representative images were shown in Fig. 4B. The correlation between vimentin expression and M1, M2 or CD8+ T cells infiltration was analyzed by assessing the immunohistochemical results. The results showed that vimentin expression was positively correlated with M1 and M2 number in lung cancer, however, negatively correlated with the ratio of M1/M2 (Fig. 4C). Meanwhile, vimentin expression was positively correlated with CD8+ T cells number in lung cancer (Fig. 4C).

However, the correlation between immune infiltrates and ECI also revealed heterogeneity between different types of cancer (Fig. 4D). As shown in Fig. 4B, a higher proportion of infiltrating immune cells are positively correlated with ECI in most cancer types, whereas only two types of cancer (CESC and UCEC) had a higher proportion of infiltrating immune cells negatively correlated with ECI.

We also investigated OV and HNSC, where a mesenchymal subtype has been identified and observed to consistently possess a relatively high fraction of M2 macrophages, and a relatively low fraction of CD8+ T cells and T helper cells in the mesenchymal subtype of cancer (Fig. 4D). To obtain an overall view of how the immune composition determines the ECI, we clustered pan-cancer tumor samples using UMAP based on the relative fraction of 22 immune subpopulations (Fig. 4E). The cluster analysis of the cellular composition of immune infiltrates revealed a clear transition from ECI_low_ tumors to ECI_high_ tumors, suggesting that ECI was determined by the cellular composition of immune infiltrates. This is consistent with our observation that the response to ICB was associated with ECI. We further divided immune cells into immunity-favoring and EMT-favoring groups, which may tip the balance toward anti-immune evasion or EMT, respectively. Characterization of the cellular composition of the immune infiltrates revealed heterogeneity of the immunity-favoring and EMT-favoring cellular profiles between different types of cancer (Fig. 4F). As shown in Fig. 4F, most of the types of cancer assessed in the present study were shown to have a high infiltration of EMT-favoring cells in the TME, whereas only two types of cancer (CESC and UCEC) had a high infiltration of immunity-favoring cells.

### Characterization of molecular and genetic features affecting the immunomodulatory crosstalk between EMT and immune evasion

**Fig. 5:**
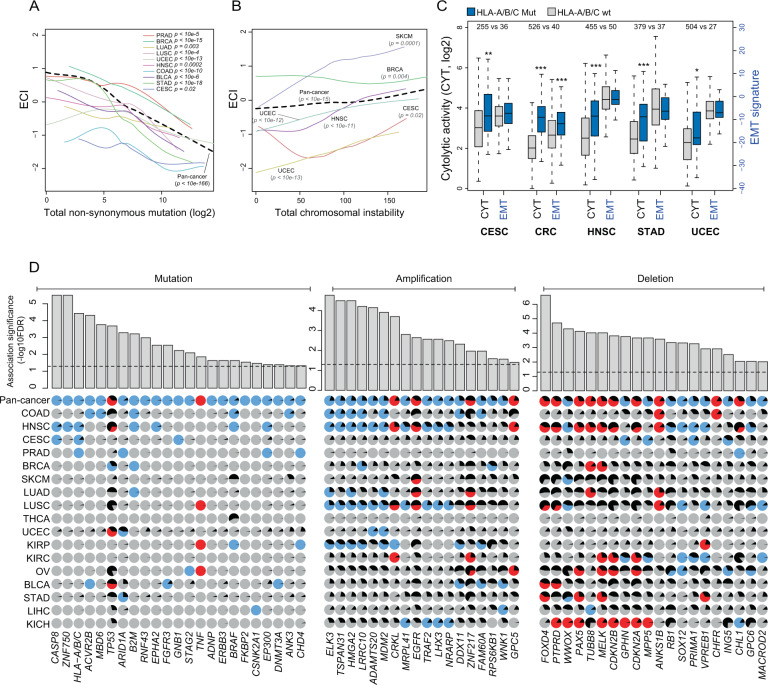
Molecular and genetic determinants of EMT and immune evasion interactions. **A** Correlation between the overall non-synonymous mutation burden and ECI in multiple types of cancer through local regression analysis. **B** Correlation between total chromosomal instability and ECI in multiple types of cancer through local regression analysis. **C** Boxplots of CYT and EMT in HLA-A/B/C genes in wild-type and mutated tumors in multiple types of cancer. Statistical analysis was performed using a Mann−Whitney *U* test. ^*^P < 0.05, ^**^P < 0.01, ^***^P < 0.001. **D** Correlation between 23 mutated, 18 amplified and 20 deleted driver genes with ECI using pan-cancer regression analysis adjusted for background mutation rate and tumor type. Positive correlation is indicated with red circles, negative with blue circles, and no correlation with gray circles. The black wedges represent the share of samples exhibiting the event.

Next, we investigated whether the molecular and genetic features of tumor cells may affect the immunomodulatory crosstalk between EMT and anti-immune evasion. It has been reported that non-synonymous mutations of genes contribute to the production of neo-antigens in tumor cells and thus may increase the infiltration of effector immune cells.^35,36^ Therefore, we first examined the relationship between CYT-EMT crosstalk and genomic features. We found that the overall rate of non-synonymous mutation was positively correlated with the CYT level (Supplementary Fig. 4A) and the fraction of immune cells negatively correlated with ECT, such as CD8 T cells, in different types of cancer (Supplementary Fig. 4B). On the contrary, the total amount of non-synonymous mutation was negatively correlated with both the EMT signature (Supplementary Fig. 4C) and the fraction of immune cells positively correlated with ECT, such as M2 macrophages (Supplementary Fig. 4D). Consistent with the above results, we found that the ECI was negatively correlated with the overall non-synonymous mutation burden in multiple types of cancer (Fig. 5A), suggesting that the mutation burden is associated with the infiltration of immune cells negatively correlated with ECT.

It has been recently reported that genome instability is linked to cancer immunity.^37^ Therefore, we examined the associations of genome instability with the interplay between EMT and immune evasion. As shown in Fig. 5B, there was a significant positive correlation between chromosomal instability and ECI in multiple types of cancer, suggesting that chromosome instability was associated with the infiltration of immune cells positively correlated ECI. As anti-immune evasion may select for tumors with genetic features that render them resistant to immune attack and are thus enriched for immune cells positively correlated ECI, we further investigated the mutations in genes involved in antigen presentation. Mutation of HLA-A/B/C genes was associated with higher CYT levels in several types of cancer, but was not always associated with a significantly higher EMT signature (Fig. 5C). Consistently, mutations in the HLA genes were positively correlated with the infiltration of CD8+ effector T cells, but there was no association with Tregs (Supplementary Fig. 4E). These results suggest that mutations in HLA genes do not increase EMT, although they may facilitate an escape from immune attack.

### Oncogenic gene alterations are associated with the immunomodulatory crosstalk between EMT and immune evasion

Tumor driver genes serve essential roles in the initiation and progression of cancer. Thus, we reasoned that alterations in oncogenic genes might be responsible for the balance between EMT and immune evasion. We focused on 373 driver genes with mutations and 200 driver genes with CNVs identified in previous pan-cancer studies.^38,39^ We implemented a pan-cancer regression analysis, adjusting for background mutation rate and tumor type, and identified 23 mutated, 18 amplified and 20 deleted driver genes that were significantly associated with ECI (Fig. 5D).

Loss of cell-cell and cell-ECM contact was necessary for EMT, but this process would induce apoptosis (termed anoikis). Thus apoptosis resistance is critical for EMT.^40^ Among these altered driver genes, *CASP8* was the most enriched gene associated with ECI in CESC and HNSC (Fig. 5D). It has been reported that *CASP8* facilitates the EMT process through enhancing the activity of Src,^41^ whereas blockage or mutation of *CASP8* results in the escape of tumor cells from immune attack.^30,42^ Additionally, alterations in four driver genes (*CDKN2A*, *BRAF*, *ERBB3* and *TRAF2*) that are significantly associated with ECI have already been described to serve essential roles in regulating apoptosis.^43–45^

Deficiency of DNA damage repair, on the one hand, would result in an accumulation of tumor neoantigens and subsequently increase the infiltration of immune cells,^46^ but on the other hand, would result in genomic instability and promote EMT.^47^ The TP53 signaling pathway serves an important role in maintaining genomic stability. Consistently, members of the TP53 signaling pathway (such as *MDM2*, *TP53* and *CDKN2A*) were found to contain driver alterations that were significantly associated with ECI (Fig. 5D). For example, *CDKN2A* has been demonstrated to inhibit EMT and stimulate cancer immunity.^48–50^ *MDM2* amplification can promote EMT and suppresses cancer immunity.^50,51^ Mutations of P53 are well known to promote EMT in various types of cancer.^52–54^ However, several studies have suggested that mutant P53 inhibits immune evasion, whereas activation of wild-type P53 overcomes immune suppression in the tumor microenvironment.^55–57^ Consistently, we observed that mutations of P53 were positively correlated with ECI (Fig. 5D). Together, these identified ECI-associated oncogenic gene alterations may act as genomic drivers of immunomodulatory crosstalk between EMT and immune evasion.

## Discussion

During the development of cancer, the interaction between cancer cells with the immune system is dynamic, and this process is called immunoediting. During the early stages of cancer progression, immune cells usually prevent tumor formation via immune surveillance; however, as tumors develop, they eventually acquire an ability to evade destruction by the immune system, and tumor-associated immune cells may even promote cancer progression.^58^ Inducing EMT in cancer cells is a significant mechanism by which tumor-associated immune cells promote cancer progression,^59^ and an innate immune response in tumor microenvironments inducing EMT has been widely acknowledged.^60^ However, studies of adaptive immune response inducing EMT are limited. In particular, there are only a few studies that have shown the roles of cytolytic immune cells in EMT of cancer cells.

It was first directly demonstrated that CD8+ T cells promote cancer relapse by inducing EMT-mediated immunoediting in cancer by Radisky’s group.^25^ They transplanted epithelial tumors expressing neo-antigens into syngeneic mice and observed T cell-dependent rejection, but subsequently, an unexpected relapse. The relapsed tumors had a mesenchymal phenotype and were negative for neo-antigens.^61,62^ Furthermore, they demonstrated that CD8+ T cells are necessary for tumor relapse. The infiltrated CD8+ T cells from mice breast tumors expressing neo-antigens were isolated and co-cultured with neu-positive tumor cells, subsequently resulting in antigen loss in the neo-positive tumor cells by inducing EMT in them and the acquisition of breast cancer stem cell properties.^25^ Recent research has shown that certain types of melanoma tumors expressing mesenchymal markers are high in cytolytic immune activity and exhibit improved patient survival,^63^ suggesting that high cytolytic activity may induce EMT in melanomas. To the best of our knowledge, the present study is the first to detect the effects of cytolytic cells on EMT pan-cancer. In contrast to their typically described protective roles, we revealed that high cytolytic activity induced EMT in the majority of the assessed solid tumors, which inhibited the favorable effects of cytolytic activity across all types of cancers.

Previous studies have suggested that infiltrated immune cells in the TME induce EMT through increased production of growth and inflammatory factors. In the present study, we detected specific growth factors and inflammatory factors of CD8+ T cells by comparing well-documented inducers of EMT produced by different immune cells, and CD8+ T cells appeared to produce more PDGFB compared with immune cells, suggesting that PDGFB may contribute to CD8+ T cell-mediated induction of EMT in the TME. It should be noted, in the present study, we investigated only well-documented EMT inducers, and there may be other undetected factors that serve equally important or even more pronounced roles in cytolytic cell-mediated induction of EMT in the TME. Furthermore, EMT-signatures do not only derive from the tumor cell, but also derive from immune cells and stromal cells, which is hard to distinguish, and therefore, further studies are required to elaborate on this. Although crosstalk between EMT and immune evasion has been investigated in this study, the causality/mechanism is not substantiated. In addition, the ECI defined here involves many genes, which makes it challenging to narrow down to specific targets/mechanisms in further studies. Finally, although the ECI was tested in public patient cohorts, validation on real tumor tissue should be done.

In summary, the present study employed a large-scale quantitative multi-omics approach to characterize the complex and dynamic immunomodulatory crosstalk between EMT and immune evasion across a broad spectrum of solid tumors. We identified potential factors associated with the balance between EMT and immune evasion. All these results and models presented here deepen our understanding of the complexity in tumors and immune and EMT interactions and may assist in providing an improved mechanistic insight for the development of novel biomarkers to predict the response to immunotherapy.

## Methods

### Data sets

We obtained relevant multi-omics data containing expression profiles, exon sequencing data and clinical data from 8200 tumors across 17 different types of solid cancer from The Cancer Genome Atlas (TCGA; tcga-data.nci.nih.gov/tcga), including urothelial bladder carcinoma (BLCA, *n* = 414), breast cancer (BRCA, *n* = 1119), cervical squamous cell carcinoma and endocervical adenocarcinoma (CESC, *n* = 306), colon adenocarcinoma (COAD, *n* = 650), head and neck squamous cell carcinoma (HNSC, *n* = 504), kidney chromophobe (KICH, *n* = 66), kidney renal clear cell carcinoma (KIRC, *n* = 542), kidney renal papillary cell carcinoma (KIRP, *n* = 291), liver hepatocellular carcinoma (LIHC, *n* = 374), lung adenocarcinoma (LUAD, *n* = 541), lung squamous cell carcinoma (LUSC, *n* = 495), ovarian serous cystadenocarcinoma (OV, *n* = 430), prostate adenocarcinoma (PRAD, *n* = 502), skin cutaneous melanoma (SKCM, *n* = 472), stomach adenocarcinoma (STAD, *n* = 420), thyroid carcinoma (THCA, *n* = 513) and uterine corpus endometrial carcinoma (UCEC, *n* = 554). Additionally, five independent immunotherapy datasets treated with immune blockade therapy (ICB) (anti-PD-1/PD-L1 or anti-CTLA4 treatment) were collected from Van Allen,^64^ Alexandra Snyder,^65^ Aleix Prat,^66^ Nadeem Riaz^67^ and Willy Hugo,^68^ which included treatment for non–small cell lung carcinoma (NSCLC), HNSC, metastatic melanoma and metastatic urothelial cancer (mUC). Additionally, 327 transcriptomes for 28 immune cell types were collected from Charoentong.^69^ Transcriptome data of cancer cells were downloaded from the Cancer Cell Line Encyclopedia (CCLE) database.^70^ Non-synonymous mutation data and genomic instability were obtained from TCGA, whereas mutations and copy number variants (CNVs) and data on driver genes in pan-cancer were obtained as previously described.^38,39^

### A quantitative model of immunomodulatory crosstalk between EMT and immune evasion

The EMT status of cancer cells was measured using EMT signature score, which is based on comparing the expression of 145 epithelial marker genes and 170 mesenchymal marker genes, as previously described.^71^ EMT score has an inbuilt cutoff of zero to identify epithelial-like (<0) and mesenchymal-like (>0) tumors. The immune evasion was assessed using the immune cytolytic activity (CYT) proposed by Rooney et al., which was quantified using the geometric mean of GZMA and PRF1 expression values.^30^ A quantitative model, termed **E**MT-**C**YT Index (ECI), was generated to describe the immunomodulatory crosstalk between EMT and immune evasion based on the standard deviations from what would be expected from the corresponding CYT score in a tumor as Eq. 1

1 $$\begin{array}{ll}{\mathrm{EMT}} = (\overline {Gene} _{mes} - \overline {Gene} _{epi})/S_{\overline {Gene} _{mes} - \overline {Gene} _{epi}},{\mathrm{CYT}} = \sqrt {GZMA \ast PRF1} ,\,{\mathrm{ECI}}\\ \quad\quad\,\,\,= {\mathrm{ln}}(\exp (\frac{{EMT - \overline {EMT} }}{{S_{EMT}}})/\exp (\frac{{CYT - \overline {CYT} }}{{S_{CYT}}}))\end{array}$$

Tumors with ECI scores >0 (termed ECI_high_ tumors) where considered to have a higher EMT signature than that expected based on the corresponding CYT level, whereas the tumors with an ECI < 0 (termed ECI_low_ tumors) where considered to have a relatively lower EMT signature than that expected based on the corresponding CYT level.

### Immune infiltration in the tumor microenvironment

We used Cibersort to evaluate the relative fraction of 22 immune cell subtypes.^72,73^ The correlation between the 22 immune cell types and ECI was analyzed using Pearson’s correlation coefficient. To visualize the extent to which the ECI was determined by the relative composition of immune cell subtypes, a unified manifold approximation and projection (UMAP) analysis was performed to reduce the 22-dimensional space to a 2-dimensional space and colored each sample based on the ECI.

### Determination of molecular and genetic features of cancer cells associated with immunomodulatory crosstalk between EMT and immune evasion

The driver mutated genes and genes with higher CNVs in pan-cancer were identified as previously described.^38,39^ To assess whether a driver gene was associated with ECI, we modeled ECI as a linear regression function of the gene’s copy number or mutation status by controlling for background mutation frequency and cancer type (encoded by 17 dummy variables).

### Immunohistochemistry assay

CD molecules and Vimentin were detected using immunohistochemistry in lung cancer tissues obtained from 30 patients with lung cancer who received tumor resection from May 2018 to September 2020 in Harbin Medical University Cancer Hospital. This study was approved by the Ethics Committee of Harbin Medical University and written informed consent was obtained from all the participants. CD68, CD16, CD163 and CD8 were used to mark macrophages, M1 macrophages, M2 macrophages and cytotoxic T lymphocyte (CTL), respectively. Vimentin is the mesenchymal marker.

In brief, the endogenous peroxidase in sections was removed by incubating with H_2_O_2_ at room temperature (RT) for 10 min after routine dewaxing and hydration. Antigens were retrieved by micro-wave heating. The sections were incubated with primary antibody against CD68 (Abcam, Cambridge, MA, USA; No. ab213363; 1:100 dilutio), CD16 (Abcam; No. ab183354; 1:200 dilution), CD163 (Abcam; No. ab182422; 1:500 dilution), CD8 (Abcam; No. ab101500; 1:800 dilution) and Vimentin (Abcam; No. ab16700; 1:200 dilution) at 4°C overnight, and then incubated with the streptavidin-biotin peroxidase-labeled secondary antibody at 37 °C for 30 min. DAB substrate (ZSGB Bio, Beijing, China) was used to show the positive result.

CD-positive cells were counted in five high power fields (HPFs) through microscopy. The average value was used to reflect the number of macrophages or CTL infiltrated in cancer tissues. The expression of Vimentin was quantified by scoring the area and intensity of staining. Staining intensity was quantified as follows: negative(0), weak,^1^ moderate,^2^ or strong.^3^ Staining area was scored according to the percentage of positive cells: none(0), <25%,^1^ 25–50%,^2^ 50–75%,^3^ or >75%.^4^ The final score was the intensity score × the area score. The results were evaluated by two pathologists in a double-blind manner.

### Statistical analyses

The enrichment of hallmark pathways across 17 different cancer types was performed by gene set enrichment analysis (GSEA), and a false discovery rate <0.05 was considered statistically significant. CYT-dependent and EMT-dependent survival analyses were analyzed using a Cox proportional hazards model. The influence of tumor mutational burden (TMB), EMT, CYT, and ECI on the response to immunotherapy was evaluated by receiver operating characteristic (ROC) curves. All statistical analyses were performed using R version 3.6.0.

### Reporting summary

Further information on research design is available in the Nature Research Reporting Summary linked to this article.

## Supplementary information

Supplementary Information

Reporting Summary

## Data Availability

The Cancer Genome Atlas (TCGA) pan-cancer data were obtained from UCSC Xena(https://gdc-hub.s3.us-east-1.amazonaws.com/download/GDC-PANCAN.htseq_fpkm-uq.tsv.gz). Immunotherapy datasets were from Van Allen,^64^ Alexandra Snyder,^65^ Aleix Prat,^66^ Nadeem Riaz^67^ and Willy Hugo.^68^ 327 transcriptomes for 28 immune cell types were collected from Charoentong.^69^ Transcriptome data of cancer cells were downloaded from the Cancer Cell Line Encyclopedia (CCLE) database (https://portals.broadinstitute.org/ccle/).^70^ Mutations and copy number variants and data on driver genes in pan-cancer were obtained from previous published studies.^38,39^
