# Supplementary material for: The pan-cancer landscape of crosstalk between epithelial-mesenchymal transition and immune evasion relevant to prognosis and immunotherapy response
Source: NPJ Precis Oncol. 2021 Jun 22;5:56. doi: 10.1038/s41698-021-00200-4 (PMC8219790; doi:10.1038/s41698-021-00200-4)
Supplement: Supplementary file 1 — Supplementary Information [file 41698_2021_200_MOESM1_ESM.pdf]

Supplementary information

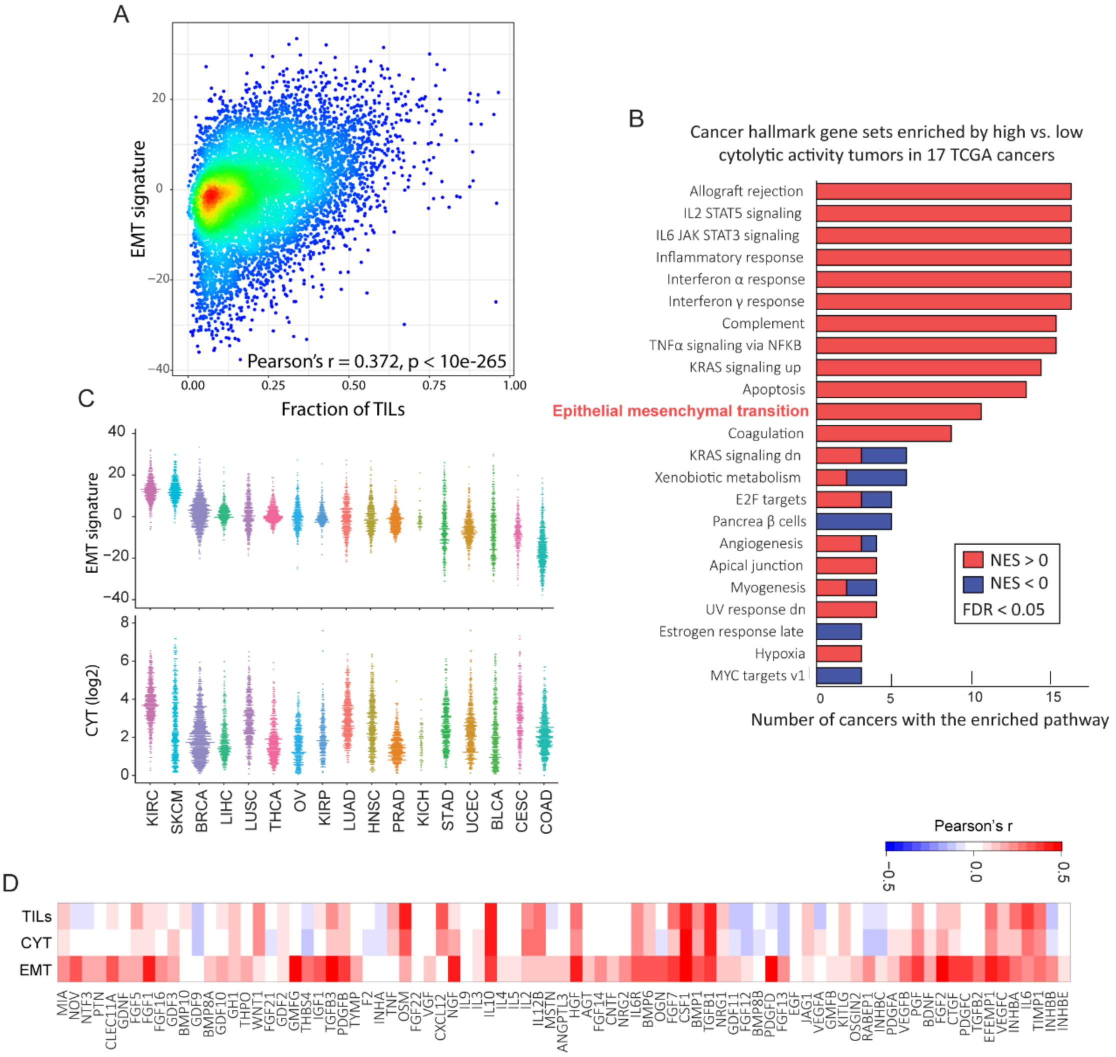

Supplementary Figure 1. Immunomodulatory crosstalk between EMT and tumor immunity. Related to Figure 1. (A) Scatter plot displaying the correlation between the fraction of TIL and EMT levels. (B) Cancer hallmark gene sets enriched by comparing CYT_{high} with CYT_{low} in the 17 types of solid cancer (false discovery rate <0.05). (C) Violin plots showing CYT and EMT levels across 17 types of solid cancer. (D) Correlation between 83 EMT-associated growth factors with TILs, CYT and EMT. CYT, cytolytic activity; EMT, epithelial-mesenchymal transition; TILs, tumor-infiltrating leukocytes

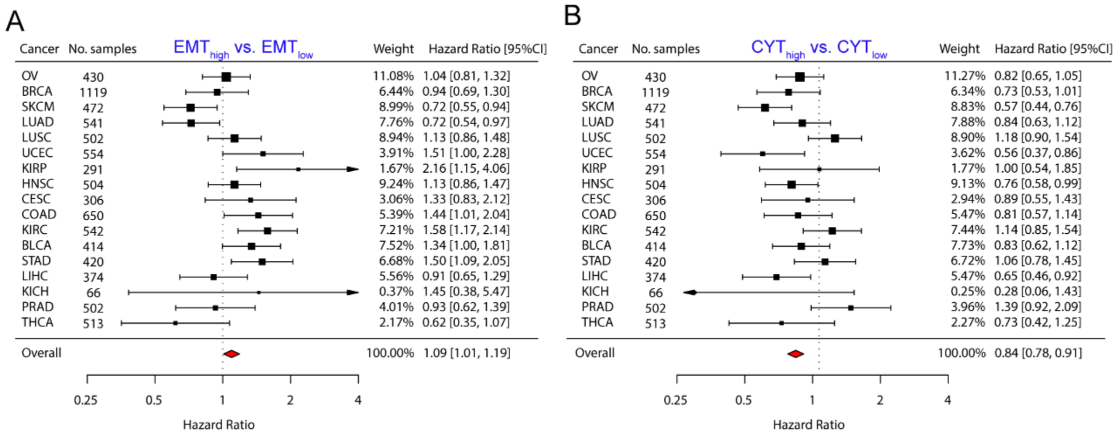

Supplementary Figure 2. Prognostic significance of EMT and CYT. Related to Figure 2.

(A) Forest plot visualizing the hazard ratios of univariate Cox proportional regression analyses of EMT levels across the 17 types of solid cancer. (B) Forest plot visualizing the hazard ratios of univariate Cox proportional regression analyses of CYT levels across the 17 types of solid cancer. The diamonds show the fixed-effects meta-analysis summary of hazard ratios across the 17 types of solid cancer. CYT, cytolytic activity; EMT, epithelial-mesenchymal transition.

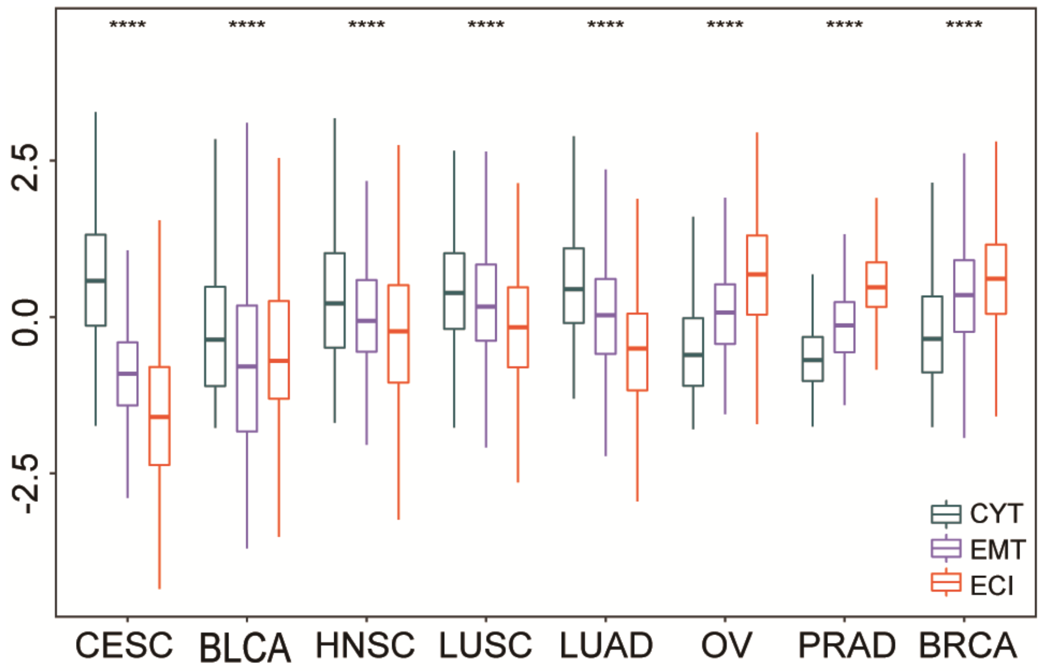

Supplementary Figure 3. Boxplot showing the distribution of ECI, CYT, EMT in cancers with high and low ICB response. Statistical analysis was performed using the Kruskal-Wallis test. **** $P < .0001$. CYT, cytolytic activity; EMT, epithelial-mesenchymal transition; ECI, EMT-CYT index.

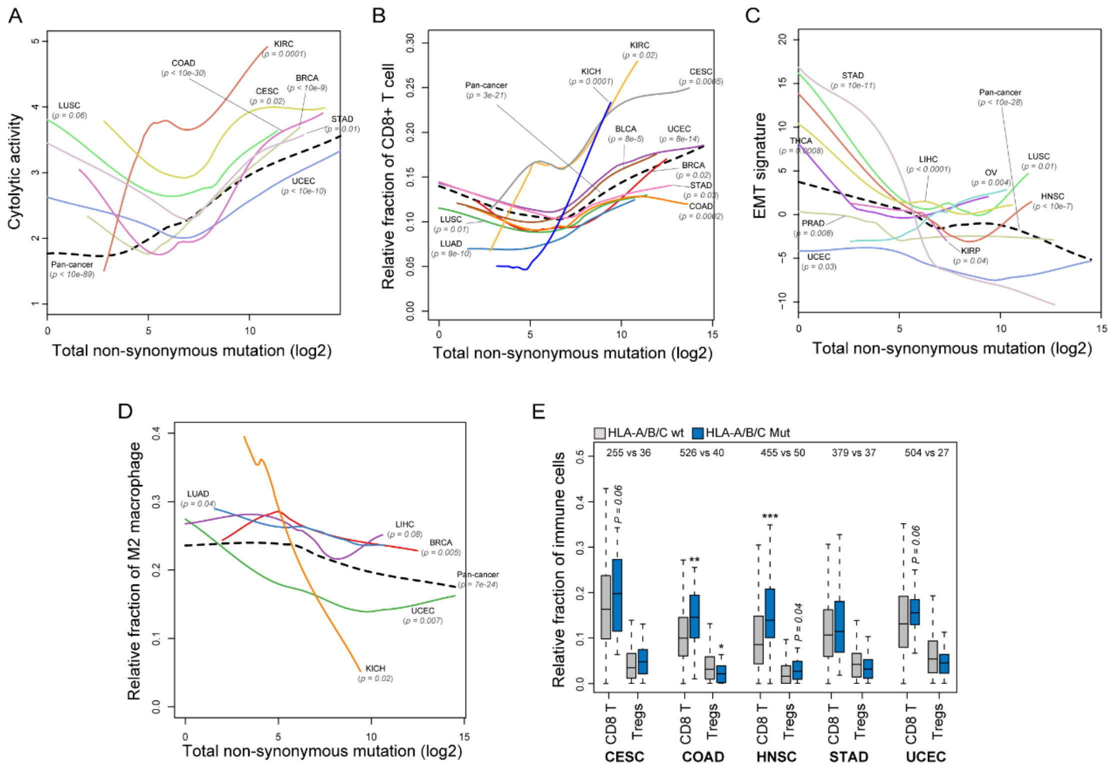

Supplementary Figure 4. Molecular and genetic determinants of EMT and tumor immunity interactions. Related to Figure 5. Local regression curves showing the significant relationships between the total non-synonymous mutation burden and (A) CYT, (B) EMT, (C) CD8+ T cell and (D) M2 macrophages in multiple types of cancer. (E) Relative infiltration fraction of CD8+ T and Tregs cells in HLA-A/B/C genes in wild-type and mutated tumors in five different types of cancer. Statistical analysis was performed using a Mann-Whitney U test. * P < .05, ** P< .01, *** P < .001.
